# An Online 3D Modeling Method for Pose Measurement under Uncertain Dynamic Occlusion Based on Binocular Camera

**DOI:** 10.3390/s23052871

**Published:** 2023-03-06

**Authors:** Xuanchang Gao, Junzhi Yu, Min Tan

**Affiliations:** 1 State Key Laboratory of Management and Control for Complex Systems, Institute of Automation, Chinese Academy of Sciences, Beijing 100190, China; 2 School of Artificial Intelligence, University of Chinese Academy of Sciences, Beijing 100049, China; 3 State Key Laboratory for Turbulence and Complex Systems, Department of Advanced Manufacturing and Robotics, Beijing Innovation Center for Engineering Science and Advanced Technology (BIC-ESAT), College of Engineering, Peking University, Beijing 100871, China

**Keywords:** 3D modeling, online, uncertain dynamic occlusion, segmentation, pose measurement

## Abstract

3D modeling plays a significant role in many industrial applications that require geometry information for pose measurements, such as grasping, spraying, etc. Due to random pose changes in the workpieces on the production line, demand for online 3D modeling has increased and many researchers have focused on it. However, online 3D modeling has not been entirely determined due to the occlusion of uncertain dynamic objects that disturb the modeling process. In this study, we propose an online 3D modeling method under uncertain dynamic occlusion based on a binocular camera. Firstly, focusing on uncertain dynamic objects, a novel dynamic object segmentation method based on motion consistency constraints is proposed, which achieves segmentation by random sampling and poses hypotheses clustering without any prior knowledge about objects. Then, in order to better register the incomplete point cloud of each frame, an optimization method based on local constraints of overlapping view regions and a global loop closure is introduced. It establishes constraints in covisibility regions between adjacent frames to optimize the registration of each frame, and it also establishes them between the global closed-loop frames to jointly optimize the entire 3D model. Finally, a confirmatory experimental workspace is designed and built to verify and evaluate our method. Our method achieves online 3D modeling under uncertain dynamic occlusion and acquires an entire 3D model. The pose measurement results further reflect the effectiveness.

## 1. Introduction

With the increasing demands for intelligent manufacturing and industrial automation, digital three-dimensional (3D) models of workpieces hold increasingly important status in production processes [1,2], such as grasping, spraying, etc., which refer to the whole 3D point cloud rather than the 2.5D point cloud in a single frame captured by the depth sensor.

3D modeling applied in factories can be performed offline or online [3,4,5]. Offline 3D modeling methods can achieve high accuracy and are often used in high precision applications, such as defect detection. However, their efficiency is low and they have ex situ processes. Online 3D modeling is often used to measure the pose of the workpiece as a standard guide [6,7,8]. For example, the pose of the workpiece is necessary for robotic spray painting, and the 3D model is an important prerequisite for pose measurement. Because random pose changes often occur during workpiece processing on the production line, online 3D modeling becomes more important for pose measurement. Moreover, an entire 3D model contains rich geometric information, which can guarantee the accuracy of pose measurement, while the 2.5D point cloud obtained from a single view is not sensitive to the shape of the workpiece, resulting in a decrease in the accuracy of the pose. Although there are many online 3D modeling methods, online 3D modeling has not been entirely determined because it is often occluded by uncertain dynamic objects, such as tools, etc. Occlusion also makes the workpiece point cloud of each frame incomplete, which will affect the accuracy and reliability of registration.

Common online 3D modeling methods can be divided into two categories. One is to arrange multiple depth sensors around the workpiece, and register point clouds collected from multiple perspectives [2,7,9,10]. Wang et al. [2] arranged multiple laser scanners around the aircraft to obtain point clouds from multiple perspectives and calculated the initial poses by detecting markers placed around the aircraft in advance. They also proposed a density-invariant area-based method to improve registration accuracy. Finally, the 3D model was used to measure the poses of the key parts of aircraft. Gao et al. [7] modeled the workpiece by registering point clouds captured by four precisely calibrated RGB-D cameras arranged around the workpiece. Bi et al. [9] also utilized multiple laser scanners and registered point clouds from multiple views to acquire the entire point clouds for different kinds of complex workpieces. Then, 3D models were utilized to estimate the relative poses to the standard position for the corresponding spraying operations. These methods are convenient and fast, but they cannot handle fixed occlusion since all sensors are fixed. Furthermore, their cost is very high and the accuracy is sensitive to the sensor calibration parameters. In many cases, sensors need to be frequently calibrated. The other online 3D modeling method is to only use one depth sensor moving around the workpiece and fuse the depth data of each frame into the global implicit surface model of the workpiece [1,11,12]. KinectFusion [11] is a typical representative, which registers the point cloud of each frame with a continuously updated model based on the truncated signed distance function voxel approach. Inspired by KinectFusion, Lin et al. [12] used a depth camera rotating around the workpiece to collect depth images and used the KinectFusion algorithm to build an entire 3D model for estimating the current pose and adjusting the robot path. These methods can achieve good performance, but their accuracy will decrease owing to occlusion. Both categories can achieve online 3D modeling, but neither can handle the possible dynamic occlusion.

Recently, many researchers have started to focus on the occlusion problems of dynamic objects to find some ways to segment dynamic objects. Existing methods can be classified into three categories: optical flow-based, point cloud clustering-based, and learning-based methods. A traditional segmentation method is optical flow [13], where the motion can be segmented by the inconsistency of feature point velocities. FlowFusion [13] applies the initial pose estimated by intensity and depth image pairs and dense optical flow of the scene obtained by the Convolutional Neural Network for optical flow using Pyramid, Warping, and Cost volume (PWC-Net) [14] to calculate the 2D scene flow generated by the movement of the objects, so as to achieve the purpose of segmenting dynamic objects. The problem is that it cannot handle large motion due to the assumption of grayscale invariance. Considering that the points on the same object have similar property, some researchers have adopted point cloud clustering methods to segment possible dynamic and static objects [15,16], which are good at mining a small set of points, the motion patterns of which are significantly different from the other parts of the point cloud. StaticFusion [15] adds K-means clustering [17] and static background segmentation of point clouds, which treats the static probability of a pixel in the current frame as a continuous variable that is jointly optimized with poses. Joint-Visual Odometry-Scene Flow (Joint-VO-SF) [16] performs K-means clustering [17] in the 3D point cloud and treats each cluster as a rigid body for pose estimation. The initial estimations of poses are used for dynamic and static object segmentation. Nevertheless, since the motion and size of objects in applications are uncertain, they are not suitable for scenarios with high uncertainty. With the development of the deep learning technique, some object detection and segmentation networks are utilized to extract dynamic objects [18,19,20]. Co-Fusion [18] applies SharpMask [21] and conditional random field-based motion segmentation to detect moving objects. Mid-Fusion [19] applies Mask Regions with Convolutional Neural Network Features (R-CNN) [22] instance segmentation and MaskFusion [23] edge refinement to detect dynamic objects and implement static dense reconstruction based on ElasticFusion [24]. PoseFusion [20] mainly aims at human life scenarios. It uses the OpenPose network [25] to identify human joints as priors to perform a minimum cut to propose human body regions and achieve background reconstruction. These methods are suitable for scenes where objects are known beforehand. However, the number of object samples in many scenarios often do not meet the training requirements of learning-based methods and the appearances of offline and online are different. In conclusion, learning-based methods rely on priors and have limited adaptability, and the first two methods are weak in terms of robustness. The above methods cannot meet the needs of 3D modeling under uncertain dynamic occlusion.

In this paper, aiming at the uncertain dynamic occlusion problem, an online 3D modeling method based on a single binocular camera is proposed. This method does not rely on prior information about objects, and can only achieve the robust segmentation of feature points and corresponding point clouds (2D-3D) by analyzing the motion patterns of adjacent frames. Through exploring the distribution of pose hypotheses calculated using 2D-3D point pairs that are obtained by multiple random sampling, 2D-3D point pairs corresponding to dense regions of the pose distribution are chosen as the segmentation results. Initial poses between adjacent frames can be acquired from these 2D-3D point pairs. On this basis, considering the incomplete point cloud of the occluded target object obtained after segmentation, an optimization method based on local constraints of covisibility regions and global loop closure is proposed to optimize the local registration and the entire 3D model. Furthermore, we design and build an experimental workspace to verify the feasibility and effectiveness of our method.

## 2. Methods

**Figure 1:**
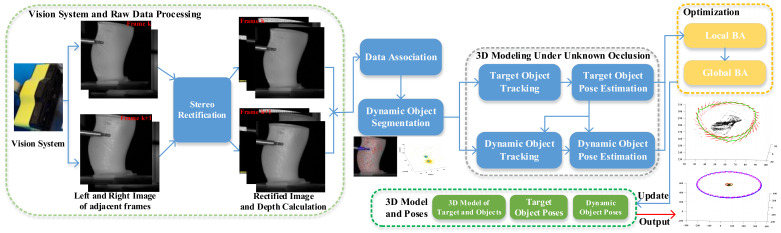
An illustration of our pipeline. (1) Vision system captures left and right images. Stereo rectification is first performed to align the epipolar lines of the left and right images by rotating two images. The depth of the points can be calculated by disparity. (2) When two consecutive frames arrive, data association is established by Grid-based Motion Statistics (GMS) and image block correlation. Then, object segmentation is carried out by random sampling and pose hypotheses clustering. (3) The segmentation results, which include initial poses and 2D-3D point pairs, are sent to the 3D modeling part. It includes the target object tracking module and the dynamic object tracking module. The target object poses (namely, camera pose) and the dynamic object motions will be estimated and optimized. (4) Finally, local BA and global BA are carried out to optimize and update these variables. The outputs of our pipeline are poses of target and dynamic objects, a 3D model, and 3D point cloud of dynamic objects.

Aimed at the task of 3D modeling under uncertain dynamic occlusion, we propose an online 3D modeling method, as shown in Figure 1. It mainly includes four steps: raw data processing and data association, dynamic objects segmentation, 3D modeling of the target object and dynamic objects, and closed-loop optimization (bundle adjustment). For the original images captured by the binocular camera, stereo rectification [26] is performed to align the epipolar lines of the left and right images. Then, the depth information of a single frame can be calculated by disparity estimation methods. After that, data association between adjacent frames is established through feature point matching, image block correlation, or auxiliary texture. That is to say, it is necessary to obtain the image point—image point correspondences (2D-2D) and the image point—3D point correspondences (2D-3D) between adjacent frames. On this basis, dynamic object segmentation is carried out to divide these 2D-3D correspondences into different objects with different motions. The initial pose of each object is estimated by these 2D-3D correspondences. Subsequently, the initial poses and points are optimized by local constraints between adjacent frames. The constraints between multiple closed-loop frames with covisibility regions are also added to the optimization. Finally, poses and 3D models of target objects and dynamic objects are acquired. In the following, uncertain dynamic object segmentation and 3D modeling based on closed-loop optimization are described in detail.

### 2.1. Object Segmentation by Clustering

Model for Dynamic Objects Segmentation. Given two consecutive images, the feature point correspondences $\left\{p_{i}\right\}$ and $\left\{q_{i}\right\}$ can be obtained first. For the dynamic object segmentation problem, we need to divide feature point correspondences so that the feature points in each divided point set conform to one motion model, the total number of motion models should be as small as possible, and the number of points in each set should be as many as possible. Therefore, the multiple dynamic object segmentation problem can be modeled as

(1) $$S_{j=1\to n_{\tau }^{*}}^{*}=\mathrm{argmin}n_{\tau }\sum_{j=1}^{n_{\tau }}\left(\sum_{p_{i}\in S_{j}}r\left(p_{i},M_{S_{j}}\right)+V\left(S_{j}\right)\right),$$

where $S_{j}$ is j-th segmented feature point set, $M_{S_{j}}$ means the motion model satisfied by points in $S_{j}$, $n_{\tau }$ denotes the number of divided objects, and $V\left(S_{j}\right)$ represents the reciprocal of the number of points in $S_{j}$. $r\left(p_{i},M_{S_{j}}\right)$ represents the reprojection error of a point $p_{i}$ on the motion model $M_{S_{j}}$. It is defined as follows:

(2) rpi,MSj=pi−1siKMSjPi2,

where $s_{i}$ is the depth of $p_{i}$, $P_{i}$ is a 3D point that can be computed by triangulation.

This model combines the complexity of segmentation, the number of inliers and the motion consistency of the object, where $n_{\tau }$ reflects the complexity of segmentation and $V\left(S_{j}\right)$ implies the number of inliers of each set. The motion consistency means that feature points belonging to the same object satisfy the same epipolar constraint:

(3) $${\left(p_{i}\right)}^{T}K^{-T}t^{⋀}RK^{-1}q_{i}=0,$$

where K refers to the camera intrinsic matrix and $t^{⋀}$ means the antisymmetric matrix of translation vector t. $\left[R,t\right]$ is the pose transformation matrix.

The best segmentation $S_{j=1\to n_{\tau }^{*}}^{*}$ can be obtained by (1). In order to solve (1), we propose a segmentation method based on cluster analysis below.

Cluster Analysis. A pose hypothesis can be obtained by one sampling in point correspondences, where one sampling represents sampling the smallest set of point pairs that are sufficient to compute a pose hypothesis. The pose hypotheses calculated from point pairs sampled on the same object must coincide in the pose parameter space. Based on this idea, cluster analysis can be achieved (1) by finding the dense regions in pose hypotheses and object segmentation can be acquired.

(1) Pose Hypothesis Calculation. To compute the pose hypothesis $M_{S_{j}}$, we consider the fundamental matrix F and the homography matrix H. We use the eight-point [27] method and the four-point [28] method to calculate F and H. Therefore, we have

(4) $$\xi =g\left(P,Q\right),$$

where $\xi \in se\left(3\right)$ is the Lie algebra form of $M_{S_{j}}$, P and Q are the sampled matching point sets. $g\left(\cdot ,\cdot \right)$ represents the algorithm for solving the pose. We need to select methods according to their applicability, limitations, and possible degradation [29]. In this paper, because the depth of points can be obtained, the perspective-n-point (PnP) method [30] is adopted to calculate the pose hypotheses. Here, the PnP algorithm requires at least four 2D-3D point pairs to compute a pose.

(2) Pose Hypotheses Clustering. Based on these pose hypotheses, the clustering algorithm is carried out. Euclidean distance in the Lie algebra coordinate system is adopted to measure the distance between two pose hypotheses.

(5) $$d_{ij}=\left\|\xi_{i},\xi_{j}\right\|.$$

Considering that the number of objects in the scene is unknown, the cluster method proposed in [31] is introduced. This method can not only automatically and accurately determine the number of cluster centers, but is also very fast. After clustering, we select the neighborhood of each cluster center as a region $\Omega_{l}$. Usually, the number of regions is the same as the number of objects. We only need to re-use the 2D-3D point pairs that calculate the pose hypotheses in each region $\Omega_{l}$ to compute a new pose, which is the pose of the object. However, due to the randomness of sampling, there may be regions made up of noise. To remove noise regions, we adopt the idea of the ordered residual kernel (ORK) [32]. We sort the number $\eta_{l}$ of poses in each region $\Omega_{l}$ in descending order $\left\{{\hat{\eta }}_{l1},{\hat{\eta }}_{l2},\cdots ,{\hat{\eta }}_{lL}\right\}$, where $\forall k:{\hat{\eta }}_{lk}\geq {\hat{\eta }}_{lk+1}$. For one region $\Omega_{lk}$ with ${\hat{\eta }}_{lk}$ pose hypotheses, the 2D-3D point pairs used to calculate pose hypotheses ξΩlki in $\Omega_{lk}$ are taken out and considered as effective points:

(6) P^Ωlki,Q^Ωlki=GξΩlki,ξΩlki∈Ωlk,

where $i=1,2,\cdots ,{\hat{\eta }}_{lk}$, P^lk=PiΩlk, Q^lk=QiΩlk. ξΩlki denotes a pose hypothesis in $\Omega_{lk}$. $G\left(\cdot \right)$ means taking out the 2D-3D point pairs $\left({\hat{P}}_{lk},{\hat{Q}}_{lk}\right)$ that calculate all hypotheses in $\Omega_{lk}$. For $\left({\hat{P}}_{lk},{\hat{Q}}_{lk}\right)$, the number of point pairs from different objects has already been significantly reduced. In other words, such an operation causes most of these 2D-3D point pairs to come from the same object. We carry out multiple sampling in $\left({\hat{P}}_{lk},{\hat{Q}}_{lk}\right)$ once more and calculate pose hypotheses. The cluster algorithm in [31] is used again to find cluster centers. The regions with only one cluster center are chosen and the regions with more than one cluster center are rejected. Finally, each remaining region corresponds to an object. The pose of each object can be calculated by 2D-3D point pairs corresponding to pose hypotheses in each region. The above process is also summarized as Algorithm 1.
**Algorithm 1.** Pose Estimation for Each Cluster Region $\Omega_{lk}$**Input:** feature points $\left({\hat{P}}_{lk},{\hat{Q}}_{lk}\right)$ taken out from $\Omega_{lk}$.**Output:** rigid body pose $\xi_{t}^{*}$ and corresponding points $\left({\hat{P}}_{t}^{*},{\hat{Q}}_{t}^{*}\right)$.1  carry out multiple sampling in $\left({\hat{P}}_{lk},{\hat{Q}}_{lk}\right)$ and obtain $\left(P_{j},Q_{j}\right)$**,**
j=1,…,N;2  $\xi_{j}\leftarrow g\left(P_{j},Q_{j}\right),j=1,2,\ldots ,N$;3  cluster $\left\{\xi_{j}\right\},j=1,2,\ldots ,N$ by [31] 4  obtain cluster centers ξcΩl, region $\Omega_{l}^{}$, poses ξkl,kl=1,…,ηlΩl, l=1,…,L;5  sort all regions in descending order according to $\eta_{l}$, $\Omega_{l1}^{},\Omega_{l2}^{},\Omega_{l3}^{},\ldots$6  **if** there is only one cluster center, that is, L=1 and is significant7    P^t,Q^t←Gξkl,kl=1,..,ηlΩl;8    $\xi_{t}^{*}\leftarrow g\left({\hat{P}}_{t},{\hat{Q}}_{t}\right)$ and select the inliers $\left({\hat{P}}_{t}^{*},{\hat{Q}}_{t}^{*}\right)$ that satisfies $\xi_{t}^{*}$;9    **return** $\xi_{t}^{*}$ and $\left({\hat{P}}_{t}^{*},{\hat{Q}}_{t}^{*}\right)$;10  **end if**11 reject the region $\Omega_{lk}$

### 2.2. 3D Modeling Based on Graph Optimization

**Figure 2:**
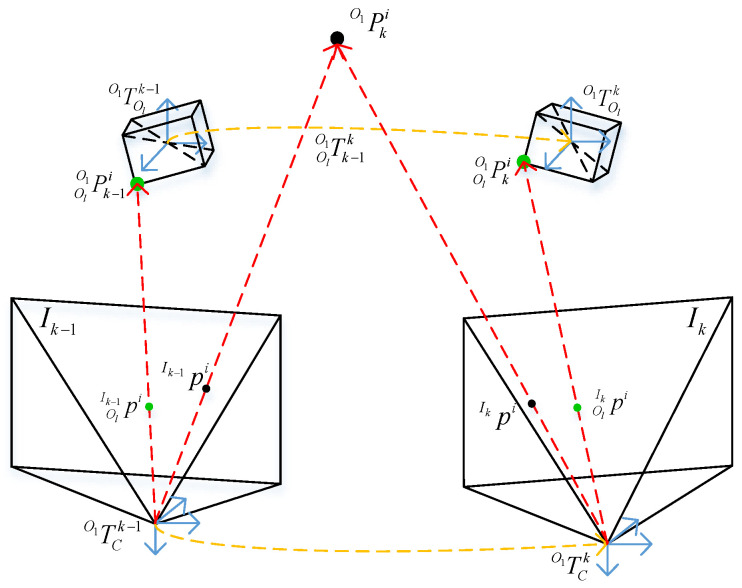
Notation and coordinate system. The big black and green circle represent the 3D point of $O_{1}$ and 3D point of $O_{l}$ in the $O_{1}$ coordinate. Small black and green circles denote the corresponding observations in images of adjacent frames. We establish the camera coordinate system in the camera optical center and the object coordinate system in the centroid of the object.

Notation. We take the coordinate system of object $O_{1}$ as the reference coordinate system and illustrate the notations in Figure 2. Each object coordinate system is established at the centroid of this object. Let TO1Ck,TO1Olk∈SE(3) denote the poses of the camera C and object $O_{l}$ in the $O_{1}$ coordinate system at the k-th frame, respectively, where k∈F, l is the l-th object, and $l=2,\cdots ,N_{L},l\neq 1$. $N_{L}$ is the total number of the objects tracked in k-th frame. In k-th frame $I_{k}$, an image feature point on $O_{1}$ is denoted by pIki=puiIk,pvi,1IkT, $i=1,2,\cdots ,N_{uv}$. It is a homogeneous coordinate representation. Similarly, an image feature point on $O_{l}$ in $I_{k}$ is represented by pOlIkj, $j=1,2,\cdots ,N_{uv}^{O_{l}}$. PO1ki and POlO1kj mean the i-th 3D point on $O_{1}$ and j-th 3D point on $O_{l}$ in the $O_{1}$ coordinate system in frame $I_{k}$, respectively. Let TO1Ck, TO1Olk be the pose transformation matrix of the camera C and object $O_{l}$ in the $O_{1}$ coordinate system at $I_{k}$, where the camera pose is the pose of the object $O_{1}$ itself. We use the notation Tk−1kOlO1 to term the motion of $O_{l}$ from $I_{k-1}$ to $I_{k}$.

Constraints and Factor Graph Establishment. After object segmentation, the 2D-3D corresponding point pairs and initial pose of each object can be obtained. Due to which object is the target object is unknown, all objects need to be estimated. Moreover, only using these initial poses cannot register the point clouds well because the point cloud of each frame is incomplete caused by occlusion. Therefore, an optimization method based on local constraints and global loop closure is introduced to acquire accurate poses.

(1) Reprojection Constraints. For a spatial 3D point PO1k−1i on $O_{1}$ in the $O_{1}$ coordinate system, according to the camera projection model, we have

(7) pIki=πTO1Ck−1PO1k−1i=KTO1Ck−1PO1k−1i1:3,

where, $\pi \left(\cdot \right)$ is the projection function, K is the 3×3 camera intrinsic matrix. ${\left[\cdot \right]}_{1:3}$ means to take the first three rows of the matrix. In other words, we take the first three components of the 4×1 column vector TO1Ck−1PO1k−1i. Given a set of 3D points PO1k−1i|k∈F,i∈Γ and its corresponding 2D image points p~Iki|k∈F,i∈Γ observed in $I_{k}$ where Γ means the index set of corresponding point pairs, we can compute the reprojection error:

(8) eIkreproji=p~Iki−πTO1Ck−1PO1k−1i,i∈Γ.

For a 3D point POlO1ki on object $O_{l}$, which can be acquired by TO1CkPOlCki, where POlCki is 3D point on $O_{l}$ in camera coordinate system, no matter how this object moves, on account of the object motion consistency, the coordinates of a 3D point POlOlki on $O_{l}$ in $O_{l}$ coordinate system do not change no matter how object $O_{l}$ moves. Therefore, we have

(9) POlOlφi=TO1Olk−1POlO1ki=TO1Olφ−1POlO1φi,φ∈F,

Here, POlOlφi denotes a 3D point of object $O_{l}$ in the object $O_{l}$ coordinate system and φ∈F can be any frame. When φ=k−1, the motion of 3D point POlOlφi can be derived by multiplying TO1Olk on the left and right sides of the second equal sign of (9):

(10) POlO1ki=TO1OlkTO1Olk−1−1POlO1k−1i=Tk−1kOlO1POlO1k−1i,

where Tk−1kOlO1 is the motion of object $O_{l}$ and can transform 3D points on object $O_{l}$ in frame k−1 to frame k in the $O_{1}$ coordinate system. On this basis, the reprojection constraints of the object $O_{l}$ can be established. Given the 2D-3D point pairs on $O_{l}$, p~OlIki|i∈ΓOl in $I_{k}$ and the 3D points POlO1k−1i|k∈F,i∈ΓOl in $I_{k-1}$ in the $O_{1}$ coordinate system, the reprojection error is

(11) eOlIkreproji=p~OlIki−πTO1Ck−1Tk−1kOlO1POlO1k−1i=p~OlIki−πTk−1kOlCPOlO1k−1i.

Here, Tk−1kOlO1=TO1CkTk−1kOlC can also be refined by optimization of TO1Ck and Tk−1kOlC.

(2) Object Motion Constraints. On the basis of object motion Tk−1kOlO1, the 3D point in $I_{k-1}$ after this motion transformation should be the same as the corresponding 3D point in $I_{k}$. Accordingly, we establish the object motion constraints:

(12) eOlO1motioni=POlO1ki−Tk−1kOlO1POlO1k−1i.

For all points on object $O_{l}$, they satisfy the same matrix Tk−1kOlO1.

(3) Smoothness Constraints. Taking into account the smoothness of the motion of the objects $O_{l}$ between adjacent camera frames, the smoothness constraint is defined as

(13) eOlsmoothk,k−1,k−2=Tk−2k−2OlO1−1Tk−1kOlO1.

That is to say, the moving speed of dynamic objects between adjacent frames is roughly the same.

All of the above constraints are added to the factor graph. The variables that need to be optimized are Ψ=TO1Ck,PO1ki,Tk−1kOlO1,POlO1ki. Then, the bundle adjustment (BA) problem can be written as

(14) Ψ*=minΨ⁡∑k∈FeIkreproji2+eOlIkreproji2+eOlO1motioni2+eOlsmoothk,k−1,k−22,

Then, (14) can be solved using the Levenberg-Marquardt method [33] to optimize the factor graph.

Here, we not only establish the above constraint relationships between adjacent frames, but also establish constraints between closed-loop frames with covisibility regions, so as to further optimize relevant variables. After optimization, 3D models and object poses can be acquired at last.

## 3. Results

Our method was verified on an experimental workspace built by ourselves. We modeled three objects under uncertain dynamic occlusion in the workspace and used the modeling results to estimate poses. The three objects were a complex surface part ($W_{a}$), a 3D printed block ($W_{b}$), and an earphone case ($W_{c}$), respectively. Their sizes were 39.52 mm×24.61 mm×38.00 mm, 40.00 mm×36.00 mm×30.47 mm, and 47.10 mm×55.24 mm×28.67 mm, respectively. In the following experiments, we only used $W_{a}$ as an example to illustrate.

### 3.1. Introduction of Confirmatory Experimental Workspace

**Figure 3:**
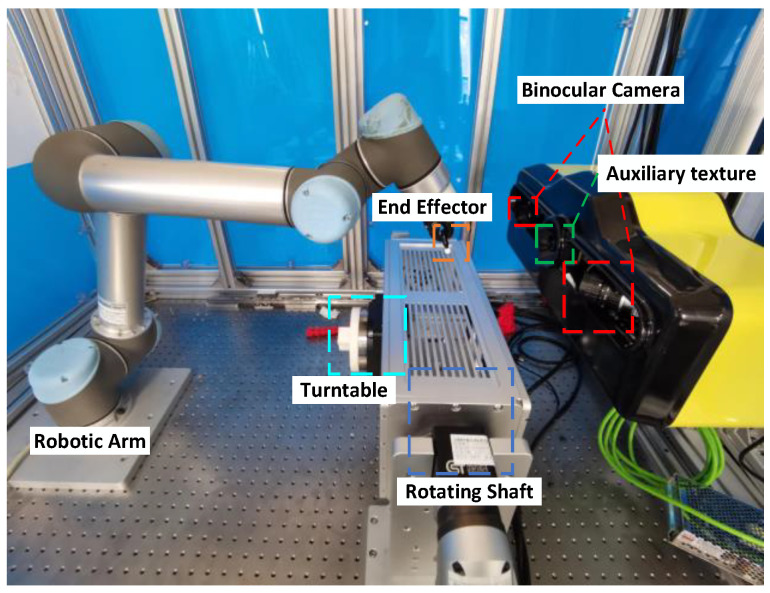
The structure of the workspace. The vision system mainly included two symmetrically arranged high-resolution cameras and an auxiliary texture device in the middle. The workpiece was placed in the white slot on the turntable. The turntable and rotating shaft could change the pose of the object. Tools could be clamped by the end effector and be moved by the robotic arm.

As shown in Figure 3, our workspace was mainly composed of a machining system, a positioner, and a vision system. The machining system consisted of a robotic arm UR5 and an end effector. Tools or other objects were clamped by the end effector and the robotic arm drove tools to perform the related operations, which could be grasping, spraying, etc. Of course, there were many kinds of tools that were not known in advance. The positioner involved a turntable and a rotating shaft. The target object was installed in the slot of the turntable. These two devices could drive the target object to change poses and they consisted of two high-precision servo motors that enabled accurate rotational control.

The vision system mainly included two high-resolution Basler cameras acA1920-50 gm (2.3 million pixels) and an auxiliary texture device. Two cameras were symmetrically arranged with a large baseline. The purpose was to improve the accuracy of depth measurements. By adjusting the focal length, the two cameras could focus on the central axis of the turntable. The auxiliary texture device was installed between two cameras. It needed to be simultaneous with the camera to provide the texture when some untextured objects were measured. After the cameras and the auxiliary texture device were installed and fixed, the calibration of the intrinsic and extrinsic parameters of the two cameras was performed. The stereo calibration method [34] was adopted to acquire these parameters. After that, stereo rectification [26] was implemented to align the epipolar lines of the left and right images. On this basis, the depth information in a single frame could be obtained by disparity or depth estimation methods. The texture of objects or auxiliary texture could be used to acquire point correspondences. The camera was also mounted on a platform controlled by servo motors and could be driven to move.

In our experiment, the camera was fixed and the target object was rotated by a positioner during modeling. To test the effectiveness of the proposed method, we kept the tool moving between the target object and the camera so that the target object was occluded throughout the whole process. The auxiliary texture was utilized to obtain the corresponding points of the left and right images. The resolution of captured images was 1920×1200. Since the background was removed, the actual maximum resolution of the image occupied by the target object and tool did not exceed 1000×1200. The vision system continuously observed the object and generated 2.5D point clouds within the field of view.

### 3.2. Demonstration of the Clustering Process

Dynamic object segmentation can be implemented by the algorithm described in Section 2.1. Before segmentation, the image corresponding points (2D-2D) of the current frame and the last frame are first obtained using the Grid-based Motion Statistics (GMS) method [35] and image block correlation [36]. Because 3D points corresponding to the image points of the last frame are known, 2D-2D point pairs can be converted into the correspondences between the image points in the current frame and the 3D points in the last frame (2D-3D point pairs). Based on these 2D-3D point pairs, the segmentation method is carried out.

**Figure 4:**
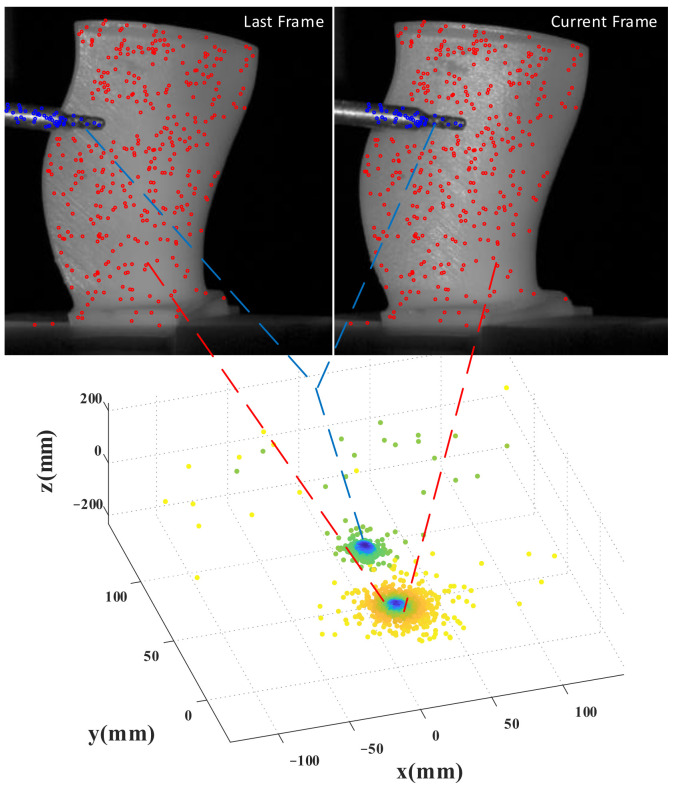
The results of segmentation and clustering. In the grayscale images, the last and current frame represent two adjacent frames and the circles indicate the matched points. The circles with different colors indicate that the feature points are located on different objects. In the three-dimensional pose parameter space, the translation vectors with the real scale are demonstrated. The shade of the color means the density is different. These two cluster centers correspond to two objects with different motions.

In order to present the clustering process more graphically, we chose the translation vector of the pose transformation to represent the pose hypothesis. The 3D pose parameter space is displayed in the 3D Cartesian coordinate system. The translations of the camera and objects have a real scale. The intermediate results of the segmentation of $W_{a}$ and the tool are shown in Figure 4. Red circles and blue circles in each grayscale image represent the points on $W_{a}$ and the tool, respectively. The black part in the grayscale image means the background has been removed. In the pose parameter space, we use shades of color to show the distribution of pose hypotheses. The darker the color (blue), the denser it is. The lighter the color (yellow), the sparser it is. It can be seen from Figure 4 that there are two obvious cluster centers, which fully reflect the existence of two objects with inconsistent motion. The 2D-3D point pairs of each object are acquired.

In our experiment, dynamic object segmentation is only performed at the beginning and when the number of points on the objects is less than a certain threshold. In other frames, the points on each object are separately tracked.

### 3.3. Modeling Results and Running Time

**Figure 5:**
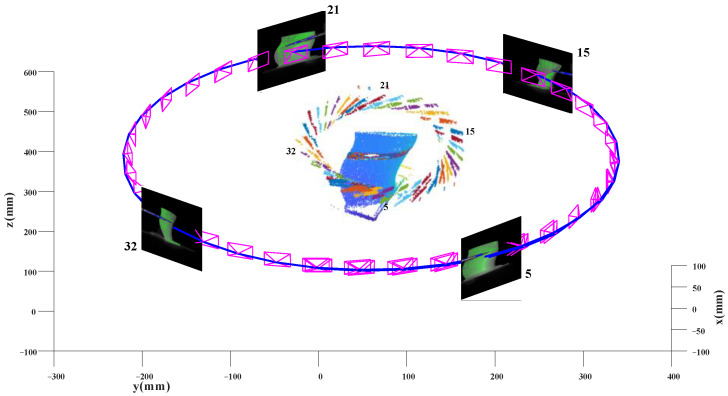
The output of our pipeline. (1) The trajectory of the camera is represented by the blue line. The magenta pyramid denotes the pose of each frame. For a clearer presentation, the images show the pictures captured by the camera at frames 5, 15, 21, and 32. In each image, the green and blue marks mean the tracked feature points on the target object and moving tool, respectively. (2) 3D model. The blue point cloud in the center of the figure is the 3D model of $W_{a}$, surrounded by the moving tool. Here, different colors show the tool observed in each frame. With the rotation of the camera and the movement of the tool, the occluded parts will be observed and modeled in other frames, as shown in the middle of the 3D model with different colors. Here, different colors represent that the occluded parts of other frames are observed in the current frame.

After object segmentation, the initial pose of each object can be acquired. Then, the factor graph optimization described in Section 2.2 is implemented to refine the poses of each object. The outputs are the camera poses and 3D models of the target object and dynamic objects, as shown in Figure 5. Usually, we take the object with the largest number of 2D-3D point pairs as object $O_{1}$. In our experiment, the target object has the largest number of point pairs, so it is object $O_{1}$, and camera poses are the poses of the target object point clouds.

In Figure 5, all results are represented in the reference coordinate system. The blue line denotes the trajectory of the camera. Each magenta pyramid denotes the 6DOF pose of each frame. For clearer presentation, we display the images of the 5th, 15th, 21th, and 32th frames captured by the camera. In each image, the green and blue marks mean the tracked feature points on the target object and tool, respectively. The 3D model of $W_{a}$ is shown in the center of Figure 5, surrounded by the 3D points of the tool. The different colors of the tool’s 3D point clouds indicate the observations of the tool in different frames. With the movement of the target object and tool, occluded parts will be observed and modeled in other frames, as shown in the middle of the target object’s 3D model with different colors. The different colors on the 3D model correspond to the colors of the tool. It demonstrates that the occluded parts in other frames are observed in the current frame. It can be seen that the occluded parts are well modeled after optimization.

We also tested the running time of optimization. The experiments were performed on an Intel Core i7-8700 3.2 GHz desktop with 24 GB RAM. For three objects $W_{a}$, $W_{b}$, $W_{c}$ to be modeled, we counted the time consumed by each important part of the program in each image frame, and calculated the average running time, as shown in the first four rows of Table 1. The last row of Table 1 gives the time for closed-loop optimization of all frames when there are closed-loop frames. In our experiment, we tracked 2000 points on the target object and 500 points on the moving tool. Local BA and global BA were implemented using the g2o [37] library.

### 3.4. Comparison Experiment (Accuracy Evaluation)

In order to evaluate the registration accuracy of the incomplete point cloud of each frame in the case of uncertain dynamic occlusion, we compared it with three methods. One was an incremental open-loop registering method. The method in [38] was utilized to calibrate the extrinsic parameters between the rotation axis and the camera. Then, point clouds were registered. The other two methods were the classic point cloud registration method Iterative Closest Point (ICP) [39] and an advanced method Correntropy-based Bidirectional Generalized Iterative Closest Point (CoBigICP) [40]. These three methods are good registration methods without moving objects. However, 3D modeling under uncertain dynamic occlusion cannot be solved. Specifically, we adopted the latter two methods to stitch point clouds, including dynamic objects. We found that they often did not converge or converged to wrong solutions due to the presence of dynamic objects, which indicated the necessity for uncertain dynamic object segmentation. Therefore, we first used our object segmentation method to segment the target object and moving tool in each frame, and then stitched the incomplete point clouds of the target object with the above three methods. Finally, the registration results of three objects $W_{a}$, $W_{b}$, $W_{c}$ obtained using the three methods and our method were compared with their ground truths (obtained by a high-precision 3D camera with a nominal accuracy of 0.02 mm). The ground truths were a set of single-frame 2.5D point clouds captured by a high accuracy 3D camera from multiple perspectives. The model obtained by each method was aligned with the ground truths. Then, registration errors (root mean squared error of the Euclidean distance of each point in a 3D model to its nearest point in ground truth) and uncertainty (standard deviation) were calculated, as shown in Table 2, where the values in brackets are the corresponding standard deviations. It can be seen from the table that the performance of our method was better than the first three methods.

**Figure 6:**
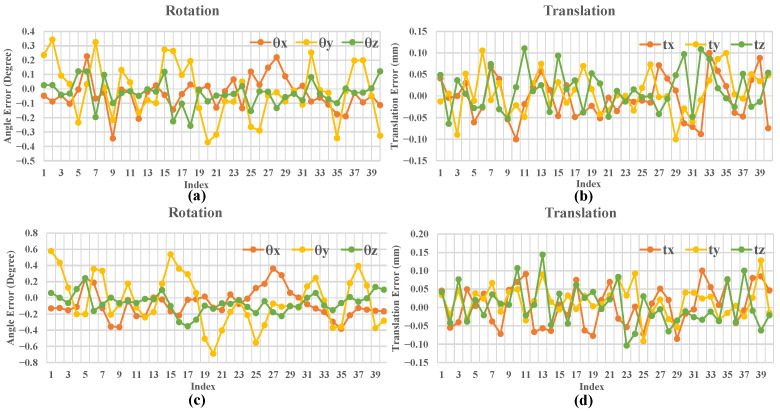
Pose errors between measured poses and their corresponding ground truths consisting of rotation errors (along the x, y, z-axes in degrees) and translation errors (along the x, y, z-axes in millimeters). We chose the pose measurement results of $T_{1}$ and $T_{3}$ to show the error variations. (**a**,**b**) are the rotation and translation errors of measurement results of $T_{1}$, respectively. (**c**,**d**) are the rotation and translation errors of measurement results of $T_{3}$.

The pose measurement results for the target object were also evaluated. The initial entire 3D model was first acquired. Then, the target object was driven by the turntable to perform pre-defined relative pose transformations. After each pose transformation, the 3D model was acquired again at this transformed position and was registered with the initial entire 3D model to estimate the relative pose transformation. The pre-defined pose transformations could be regarded as ground truths of the pose transformations between two positions. Here, we provide three pre-defined relative pose transformations $T_{k}=\left[\theta_{k},t_{k}\right]$ in the camera coordinate system where k=1,2,3, as shown by the ground truths (GT) in Table 3, where θk=θkx,θky,θkz denotes the Euler’s angles about the xyz-axes (measured in degrees) and tk=tkx,tky,tkz means translation (measured in millimeters). These three pre-defined pose relative transformations $T_{1}$, $T_{2}$, $T_{3}$, are $T_{1}=\left[{0^{{}^{\circ}},10}^{{}^{\circ}},0^{{}^{\circ}},-10,0,50\right]$, $T_{2}=\left[{0^{{}^{\circ}},-10}^{{}^{\circ}},0^{{}^{\circ}},0,0,-50\right]$, and $T_{3}=\left[{0^{{}^{\circ}},20}^{{}^{\circ}},0^{{}^{\circ}},-20,0,80\right]$, respectively. At each pre-defined pose, we performed multiple 3D modeling and measure relative pose transformations. In this experiment, our expected translation error and angle error were within 0.1 mm and 0.4 degrees, respectively. We selected the pose measurement results of $W_{a}$ corresponding to pre-defined poses $T_{1}$ and $T_{3}$, and showed the error of each measurement value compared to the ground truth in Figure 6. Figure 6a,b is the rotation and translation errors corresponding to $T_{1}$. Figure 6c,d is the rotation and translation errors corresponding to $T_{3}$. It can be seen that the angle errors are within 0.4 degrees, and translation errors are within 0.1mm, but there are a few results that exceed these values. Our method performed well. The pose measurement results of the three target objects $W_{a}$, $W_{b}$, $W_{c}$ at pre-defined position transformed by $T_{k}$ are given in the form of statistical values, as shown in Table 3. In Table 3, $\overset{-}{\theta_{k}}$ and $\sigma_{\theta_{k}}^{}$ denote the average and standard deviation (uncertainty) of measured Euler angles. ${Med}_{\theta_{k}}$ includes the median values of $\theta_{x},\theta_{y}$, and $\theta_{z}$ in the measured results. ${\left|∆\theta_{k}\right|}_{max}$ and ${\left|∆\theta_{k}\right|}_{mean}$ are the maximum and mean of the absolute errors of $\theta_{x},\theta_{y}$, and $\theta_{z}$ in the measured results, where $∆\theta_{k}=\tilde{\theta_{k}}-\theta_{k}^{gt}$, $\tilde{\theta_{k}}$, and $\theta_{k}^{gt}$ are the measurement value and ground truth, respectively. Similarly, $\overset{-}{t_{k}}$ and $\sigma_{t_{k}}^{}$ denote the average and standard deviation (uncertainty) of measured translations. ${Med}_{t_{k}}$ includes the median values of the $t_{x},t_{y}$, and $t_{z}$ in the measured results. ${\left|∆t_{k}\right|}_{max}$ and ${\left|∆t_{k}\right|}_{mean}$ are the maximum and mean of the absolute errors of $t_{x},t_{y}$, and $t_{z}$ in the measured results, where $∆t_{k}=\tilde{t_{k}}-t_{k}^{gt}$, $\tilde{t_{k}}$, and $t_{k}^{gt}$ are the measurement value and ground truth. As can be seen from Table 3, the average values of pose measurements are very close to the ground truths. Although the maximum absolute errors are large, it can be seen from the standard deviations, median values, and mean absolute errors that the measurement results are relatively stable.

**Figure 7:**
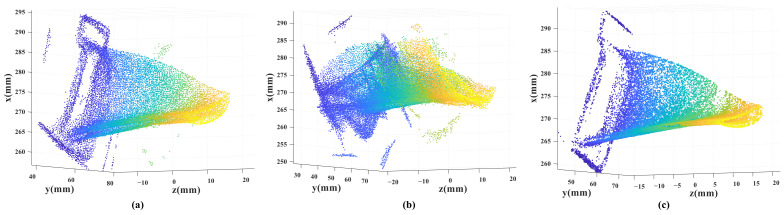
3D modeling results obtained by three methods. (**a**) is the result of the incremental open-loop registering method. The object structure cannot be recovered in (**b**) because of the disadvantages of ICP. Our method performs well in (**c**).

In order to more vividly show the registration results of the incremental open-loop registering method, ICP, and our method, we demonstrate the results of the $W_{a}$ in Figure 7. Figure 7a is the result of the incremental open-loop registering method. Due to accumulated errors, the 3D model does not close well. It can be seen there is a gap on the edge of the 3D model. The registration result of the ICP method is displayed in Figure 7b. ICP does not perform well. The possible reason is that $W_{a}$ is a complex curved structure and the ICP method tends to converge to a local optimum. As can be seen from the figure, the accumulation of errors in the registering process makes it difficult for the later point clouds to register well with the previous point clouds. Our method performs well, as shown in Figure 7c.

## 4. Discussion

In this paper, we propose a segmentation method based on motion consistency constraints without any prior knowledge, which only achieves robust segmentation by analyzing the motion patterns of adjacent frames. The demonstration experiment of the clustering process fully reflects that pose hypotheses calculated from point pairs sampled on the same object must coincide in the pose parameter space. The number of clustering centers represents the number of possible dynamic objects with an inconsistent motion. Our method does not require object samples for training compared with deep learning-based methods, and it is insensitive to the size and motion of moving objects compared with the point cloud clustering-based and optical-flow-based methods. It can meet the needs of 3D modeling under uncertain dynamic occlusion. The experiments also further emphasize the importance of dynamic object segmentation. ICP and other methods are difficult to register point clouds without segmenting dynamic objects. For the registering of incomplete point clouds, we propose an optimization method based on local constraints and global loop closure constraints. Table 2 and Figure 7 show that the optimization method plays an important role in obtaining the entire 3D model. It can effectively improve the accuracy of 3D modeling, while it will increase the calculation and time cost. As the number of feature points increases, the calculation speed decreases. Finally, the pose measurement experiment shows that the rich geometric information of the entire 3D model makes the pose measurement more accurate and reflects the accuracy of 3D modeling results.

## 5. Conclusions

Aimed at the task of 3D modeling under uncertain dynamic occlusion, we propose an online 3D modeling method for pose measurements under uncertain dynamic occlusion. Firstly, in order to solve the uncertain dynamic object occlusion problem, we propose a dynamic object segmentation method based on motion consistency constraint without any prior knowledge about the objects, which segments the objects by random sampling and pose hypotheses clustering. Then, the initial pose and 2D-3D point pairs of each object between two adjacent frames can be obtained. After that, because the incomplete point cloud of the target object of each frame needs to be registered and inspired by the constraint relationships of the covisibility regions, an optimization method based on local constraints of overlapping view regions and global loop closure is introduced to optimize the registration of adjacent frames and the entire 3D model. Furthermore, constraints between global closed-loop frames are also established to refine the results. Finally, based on the proposed framework and method, we design and build a confirmatory experimental workspace. The experiments are carefully carried out in this workspace to test and verify the performance of our method. For the modeling results of three objects, our method outperforms three other classical methods. The 3D model is utilized to measure the poses, and the poses are further compared with the ground truths. The performance of our method meets the expected requirements. 

In this paper, we mainly focus on improving the optimization speed and solving the object reflection problem. Optimization will slow down as the number of points increases. Graphics Processing Unit (GPU) acceleration and the filter-based method are considered to address this problem. Reflection is also a challenging problem in computer vision. At present, traditional methods cannot solve this problem well. We intend to introduce a learning-based technique to deal with this.

## Figures and Tables

**Table 1:** Runtime (millisecond) statistics for important parts of 3D modeling processes for three objects $W_{a}$, $W_{b}$, $W_{c}$.

	$W_{a}$	$W_{b}$	$W_{c}$
Camera Tracking and Pose Optimization	16.32	40.46	33.83
Dynamic Object Tracking	1.27	1.31	1.07
Dynamic Object Pose Estimation and Optimization	19.34	17.10	8.90
Update	11.96	18.11	22.69
Local Bundle Adjustment (In Sliding Window)	21.77	37.79	55.23
Closed-loop Optimization (All Frames from the Loop)	855.72	1432.44	2060.28

**Table 2:** Registration error (mm) and uncertainty (standard deviation, in brackets) of three objects $W_{a}$, $W_{b}$, $W_{c}$ compared with three registration methods.

	$W_{a}$	$W_{b}$	$W_{c}$
Incremental Open-loop Registering Method	0.22 (7.81 × 10^−2^)	0.35 (0.27)	0.18 (7.55 × 10^−2^)
ICP	0.54 (0.27)	0.32 (0.21)	0.19 (9.70 × 10^−2^)
CoBigICP [40]	0.13 (4.58 × 10^−2^)	0.18 (5.39 × 10^−2^)	0.11 (5.74 × 10^−2^)
Ours	0.06 (3.03 × 10^−2^)	0.07 (2.70 × 10^−2^)	0.08 (2.96 × 10^−2^)

**Table 3:** Averages, standard deviations, median values, maximum absolute errors, and mean absolute errors of pose measurement results in the camera coordinate system. $T_{1}$, $T_{2}$, $T_{3}$ are three pre-defined relative pose transformations. The 3D modeling is carried out at the initial position and the positions transformed by these three pre-defined relative pose transformation matrixes. Pose transformations between two positions can be measured by these 3D models and compared with the ground truths $T_{1}$, $T_{2}$, $T_{3}$.

Measured Poses		$W_{a}$	$W_{b}$	$W_{c}$	GT
$T_{1}$	$\overset{-}{\theta_{1}}$	$\left({-0.03}^{{}^{\circ}},9.99^{{}^{\circ}},-0.03^{{}^{\circ}}\right)$	$\left({-0.06}^{{}^{\circ}},10.03^{{}^{\circ}},-0.01^{{}^{\circ}}\right)$	$\left({{-0.02}^{{}^{\circ}},9.97}^{{}^{\circ}},{-0.03}^{{}^{\circ}}\right)$	$\left({0^{{}^{\circ}},10}^{{}^{\circ}},0^{{}^{\circ}}\right)$
	$\sigma_{\theta_{1}}^{}$	(0.11, 0.19, 0.09)	(0.19, 0.15, 0.19)	(0.10, 0.20, 0.09)	-
	${Med}_{\theta_{1}}$	$\left({-0.03}^{{}^{\circ}},9.98^{{}^{\circ}},-0.03^{{}^{\circ}}\right)$	$\left({-0.07}^{{}^{\circ}},10.02^{{}^{\circ}},-0.06^{{}^{\circ}}\right)$	$\left({{-0.02}^{{}^{\circ}},9.97}^{{}^{\circ}},{-0.02}^{{}^{\circ}}\right)$	-
	${\left|∆\theta_{1}\right|}_{max}$	$\left(0.35^{{}^{\circ}},0.37^{{}^{\circ}},0.26^{{}^{\circ}}\right)$	$\left(0.85^{{}^{\circ}},0.73^{{}^{\circ}},0.49^{{}^{\circ}}\right)$	$\left({0.31^{{}^{\circ}},0.45}^{{}^{\circ}},0.26^{{}^{\circ}}\right)$	-
	${\left|∆\theta_{1}\right|}_{mean}$	$\left(0.09^{{}^{\circ}},0.15^{{}^{\circ}},0.07^{{}^{\circ}}\right)$	$\left(0.13^{{}^{\circ}},0.09^{{}^{\circ}},0.15^{{}^{\circ}}\right)$	$\left({0.08^{{}^{\circ}},0.16}^{{}^{\circ}},0.07^{{}^{\circ}}\right)$	-
	$\overset{-}{t_{1}}$	(−10.00, 0.01, 50.01)	(−9.99, 0.01, 50.02)	(−9.98, 0.02, 50.01)	(−10, 0, 50)
	$\sigma_{t_{1}}^{}$	(0.05, 0.04, 0.04)	(0.05, 0.05, 0.04)	(0.05, 0.05, 0.06)	-
	${Med}_{t_{1}}$	(−10.01, 0.001, 50.01)	(−9.97, 0.02, 50.03)	(−9.99, 0.01, 50.03)	-
	${\left|∆t_{1}\right|}_{max}$	(0.10, 0.11, 0.11)	(0.16, 0.12, 0.11)	(0.15, 0.13, 0.14)	-
	${\left|∆t_{1}\right|}_{mean}$	(0.04, 0.03, 0.04)	(0.04, 0.05, 0.03)	(0.04, 0.04, 0.05)	-
$T_{2}$	$\overset{-}{\theta_{2}}$	$\left({{-0.01}^{{}^{\circ}},-9.96}^{{}^{\circ}},0.01^{{}^{\circ}}\right)$	$\left({0.02^{{}^{\circ}},-9.98}^{{}^{\circ}},0.04^{{}^{\circ}}\right)$	$\left(0.01^{{}^{\circ}},{-9.98}^{{}^{\circ}},0.04^{{}^{\circ}}\right)$	$\left({0^{{}^{\circ}},-10}^{{}^{\circ}},0^{{}^{\circ}}\right)$
	$\sigma_{\theta_{2}}^{}$	(0.12, 0.11, 0.19)	(0.11, 0.21, 0.09)	(0.12, 0.22, 0.09)	-
	${Med}_{\theta_{2}}$	$\left({{-0.03}^{{}^{\circ}},-9.95}^{{}^{\circ}},0.07^{{}^{\circ}}\right)$	$\left({0.01^{{}^{\circ}},-9.97}^{{}^{\circ}},0.03^{{}^{\circ}}\right)$	$\left(0.03^{{}^{\circ}},{-9.96}^{{}^{\circ}},0.03^{{}^{\circ}}\right)$	-
	${\left|∆\theta_{2}\right|}_{max}$	$\left({0.44^{{}^{\circ}},0.31}^{{}^{\circ}},0.52^{{}^{\circ}}\right)$	$\left({0.31^{{}^{\circ}},0.41}^{{}^{\circ}},0.26^{{}^{\circ}}\right)$	$\left(0.29^{{}^{\circ}},0.64^{{}^{\circ}},0.24^{{}^{\circ}}\right)$	-
	${\left|∆\theta_{2}\right|}_{mean}$	$\left({0.08^{{}^{\circ}},0.09}^{{}^{\circ}},0.14^{{}^{\circ}}\right)$	$\left({0.08^{{}^{\circ}},0.16}^{{}^{\circ}},0.07^{{}^{\circ}}\right)$	$\left(0.09^{{}^{\circ}},0.17^{{}^{\circ}},0.07^{{}^{\circ}}\right)$	-
	$\overset{-}{t_{2}}$	(0.01, 0.03, −49.97)	$\left(0.01,-0.01,-49.98\right)$	$\left(-0.01,-0.02,-50.02\right)$	(0, 0, −50)
	$\sigma_{t_{2}}^{}$	(0.04, 0.05, 0.05)	(0.05, 0.04, 0.07)	(0.04, 0.04, 0.06)	-
	${Med}_{t_{2}}$	(0.01, 0.02, −49.98)	(0.03, 0.01, −49.97)	$\left(-0.01,-0.01,-50.01\right)$	-
	${\left|∆t_{2}\right|}_{max}$	(0.09, 0.16, 0.15)	(0.15, 0.12, 0.15)	(0.10, 0.10, 0.11)	-
	${\left|∆t_{2}\right|}_{mean}$	(0.03, 0.04, 0.05)	(0.05, 0.03, 0.04)	(0.03, 0.04, 0.06)	-
$T_{3}$	$\overset{-}{\theta_{3}}$	$\left(-{0.07^{{}^{\circ}},19.97}^{{}^{\circ}},-0.06^{{}^{\circ}}\right)$	$\left({-0.12}^{{}^{\circ}},20.05^{{}^{\circ}},0.01^{{}^{\circ}}\right)$	$\left({-0.05}^{{}^{\circ}},19.95^{{}^{\circ}},{-0.07}^{{}^{\circ}}\right)$	$\left({0^{{}^{\circ}},20}^{{}^{\circ}},0^{{}^{\circ}}\right)$
	$\sigma_{\theta_{3}}^{}$	(0.16, 0.31, 0.12)	(0.23, 0.18, 0.33)	(0.16, 0.23, 0.11)	-
	${Med}_{\theta_{3}}$	$\left({{-0.12}^{{}^{\circ}},19.91}^{{}^{\circ}},{-0.06}^{{}^{\circ}}\right)$	$\left({-0.15}^{{}^{\circ}},20.03^{{}^{\circ}},{-0.12}^{{}^{\circ}}\right)$	$\left({-0.06}^{{}^{\circ}},19.90^{{}^{\circ}},{-0.07}^{{}^{\circ}}\right)$	-
	${\left|∆\theta_{3}\right|}_{max}$	$\left({0.38^{{}^{\circ}},0.69}^{{}^{\circ}},0.35^{{}^{\circ}}\right)$	$\left(0.61^{{}^{\circ}},0.88^{{}^{\circ}},0.94^{{}^{\circ}}\right)$	$\left(0.36^{{}^{\circ}},0.77^{{}^{\circ}},0.35^{{}^{\circ}}\right)$	-
	${\left|∆\theta_{3}\right|}_{mean}$	$\left({0.15^{{}^{\circ}},0.26}^{{}^{\circ}},0.10^{{}^{\circ}}\right)$	$\left(0.20^{{}^{\circ}},0.12^{{}^{\circ}},0.26^{{}^{\circ}}\right)$	$\left(0.13^{{}^{\circ}},0.27^{{}^{\circ}},0.10^{{}^{\circ}}\right)$	-
	$\overset{-}{t_{3}}$	(−19.99, 0.02, 80.01)	(−20.01, −0.01, 79.99)	(−20.02, −0.02, 79.99)	(−20, 0, 80)
	$\sigma_{t_{3}}^{}$	(0.06, 0.04, 0.05)	(0.05, 0.04, 0.04)	(0.05, 0.03, 0.04)	-
	${Med}_{t_{3}}$	(−19.98, 0.02, 79.99)	(−20.01, −0.02, 80.00)	(−20.02, −0.02, 79.98)	-
	${\left|∆t_{3}\right|}_{max}$	(0.10, 0.12, 0.14)	(0.11, 0.09, 0.13)	(0.12, 0.10, 0.09)	-
	${\left|∆t_{3}\right|}_{mean}$	(0.05, 0.04, 0.07)	(0.04, 0.04, 0.06)	(0.04, 0.05, 0.03)	-

## Data Availability

Not applicable.
